# LRRK2 kinase plays a critical role in manganese-induced inflammation and apoptosis in microglia

**DOI:** 10.1371/journal.pone.0210248

**Published:** 2019-01-15

**Authors:** Judong Kim, Edward Pajarillo, Asha Rizor, Deok-Soo Son, Jayden Lee, Michael Aschner, Eunsook Lee

**Affiliations:** 1 Department of Pharmaceutical Sciences, College of Pharmacy, Florida A&M University, Tallahassee, Florida, United States of America; 2 Department of Biochemistry and Cancer Biology, Meharry Medical College, Nashville, Tennessee, United States of America; 3 Department of Speech, Language & Hearing Sciences, Boston University, Boston, Massachusetts, United States of America; 4 Department of Molecular Pharmacology, Albert Einstein College of Medicine, Bronx, New York, United States of America; Universita degli Studi di Padova, ITALY

## Abstract

Long-term exposure to elevated levels of manganese (Mn) causes manganism, a neurodegenerative disorder with Parkinson’s disease (PD)-like symptoms. Increasing evidence suggests that leucine-rich repeat kinase 2 (LRRK2), which is highly expressed in microglia and macrophages, contributes to the inflammation and neurotoxicity seen in autosomal dominant and sporadic PD. As gene-environment interactions have emerged as important modulators of PD-associated toxicity, LRRK2 may also mediate Mn-induced inflammation and pathogenesis. In this study, we investigated the role of LRRK2 in Mn-induced toxicity using human microglial cells (HMC3), LRRK2-wild-type (WT) and LRRK2-knockout (KO) RAW264.7 macrophage cells. Results showed that Mn activated LRRK2 kinase by phosphorylation of its serine residue at the 1292 position (S1292) as a marker of its kinase activity in macrophage and microglia, while inhibition with GSK2578215A (GSK) and MLi-2 abolished Mn-induced LRRK2 activation. LRRK2 deletion and its pharmacological inhibition attenuated Mn-induced apoptosis in macrophages and microglia, along with concomitant decreases in the pro-apoptotic Bcl-2-associated X (Bax) protein. LRRK2 deletion also attenuated Mn-induced production of reactive oxygen species (ROS) and the pro-inflammatory cytokine TNF-α. Mn-induced phosphorylation of mitogen-activated protein kinase (MAPK) p38 and ERK signaling proteins was significantly attenuated in LRRK2 KO cells and GSK-treated cells. Moreover, inhibition of MAPK p38 and ERK as well as LRRK2 attenuated Mn-induced oxidative stress and cytotoxicity. These findings suggest that LRRK2 kinase activity plays a critical role in Mn-induced toxicity via downstream activation of MAPK signaling in macrophage and microglia. Collectively, these results suggest that LRRK2 could be a potential molecular target for developing therapeutics to treat Mn-related neurodegenerative disorders.

## Introduction

Manganese (Mn) is an essential trace element in the body, where it serves as a critical cofactor of several enzymes such as glutamine synthase and superoxide dismutase in the development process, metabolism and antioxidant systems [1]. However, chronic exposure to elevated levels of Mn leads to its accumulation in the basal ganglia, particularly the globus pallidus, leading to a neurological disorder referred to as manganism [2, 3]. The clinical symptoms of manganism manifest similarly to Parkinson’s disease (PD) and characterized by motor impairment, psychiatric disturbances and cognitive deficits [3, 4]. This sequalae may be associated with the Mn-induced loss of dopaminergic neurons in the substantia nigra [5–7].

While the pathological symptoms of manganism are well established, the cellular and molecular mechanisms of Mn-induced neurotoxicity are not completely understood. It has been reported that Mn induces mitochondrial dysfunction, inflammation, oxidative stress and glutamate excitotoxicity both in *in vitro* and *in vivo* experimental settings [8–11]. Moreover, Mn causes dysfunction of astrocytic glutamate transporters by decreasing the expression of astrocytic glutamate transporters GLT-1 and GLAST at the transcriptional level through activation of the transcription factor yin yang 1 (YY1) [12–14].

A number of studies of rodent and non-human primates have attributed Mn-induced injury to neuroinflammation and increased expression of pro-inflammatory cytokines, such as tumor necrosis factor-alpha (TNF-α) and interleukin 1-beta (IL-1β) [4, 15]. These pro-inflammatory cytokines are primarily produced by glial cells in response to activation by environmental toxicants, such as Mn [16]. Notably, inflammation is frequently identified as a contributing factor in multiple neurodegenerative diseases including multiple sclerosis, Alzheimer’s disease (AD), amyotrophic lateral sclerosis (ALS), PD and manganism [17–19]. However, the mechanisms underlying these inflammatory effects and the role of glial cells, particularly microglia, remain to be established.

Microglia are highly specialized macrophages responsible for scavenging debris and mounting innate immune defense in the central nervous system (CNS) [20], playing an important role in the brain’s response to toxic insults. Although microglia act as the resident brain macrophages, prolonged activation of microglia can lead to overproduction of harmful molecules such as reactive oxygen species (ROS) and pro-inflammatory cytokines [21], resulting in deleterious cellular effects and often neuronal death [22]. Environmental toxicants have long been associated with microglial inflammation and neurodegeneration [23, 24]. Moreover, activation of signaling pathways, including nuclear factor kappa B (NF-κB) and mitogen-activated kinase (MAPK), appear to play a role in Mn-induced inflammatory gene expression in microglia [23, 25, 26]. Microglia-mediated neuroinflammation is also implicated in the progression of many neurodegenerative diseases, including AD and PD [27, 28].

Although the majority of cases are sporadic, several genes have been implicated in the development of familial PD [29]. Particularly, autosomal dominant mutations in leucine-rich repeat kinase 2 (LRRK2) are associated with sporadic PD (accounting for 1–2% of all sporadic PD cases) and a familial form of PD (13% of all familial PD cases) [30]. Patients with LRRK2 mutations develop late-onset disease, with symptoms indistinguishable from idiopathic PD patients [31]. LRRK2 is a large multi-domain protein, composed of a leucine-rich domain (LRR), a Roc GTPase domain, a carboxy-terminal of Ras (COR) domain, a kinase domain and a WD40 domain at the carboxy-terminal [31, 32]. Among all the identified LRRK2 pathological mutations, G2019S, which is located in the kinase domain of LRRK2, is the most frequent mutation in both familial and sporadic PD cases [33]. The G2019S mutation located in the kinase domain leads to increased LRRK2 autophosphorylation and kinase activity [34–36] and, intriguingly, abnormally high LRRK2 kinase activity has been found to be toxic to neurons [34, 37]. Other pathogenic LRRK2 mutations such as I2020T and R1441C/G/H have also shown to enhance LRRK2 kinase activity [35, 38].

The biological function of LRRK2 remains unclear, but the presence of both a GTPase and kinase domain suggest a role in intracellular signaling [39]. LRRK2 regulates neurite morphology in rodent and human neuronal cell lines [40, 41]. LRRK2 has also been associated with vesicular trafficking [42], cytoskeletal dynamics [43], mitochondrial function [44], apoptosis [16] and autophagy [45]. In recent years, LRRK2 has been implicated in the inflammatory process, as LRRK2 is highly expressed in immune cells, particularly in microglia and macrophages [46]. Higher LRRK2 basal levels were shown in cultured microglial cells than in neuronal cells [47], suggesting a critical role of LRRK2 in microglial functions, including inflammation [48] and phagocytosis [49]. Moreover, LRRK2-associated PD patients showed elevated levels of pro-inflammatory serum markers such as interleukin-12-p40, indicating the involvement of LRRK2 in inflammatory processes [50]. LRRK2 has also been shown to induce inflammation by dysregulating the NF-κB pathway in cultured microglial cells [51].

Growing evidence implicates both genetic and environmental factors in the development of neurodegenerative disorders such as PD and manganism [52]. This pathogenesis is likely mediated by the microglial inflammatory response, but whether or not environmental toxicants such as Mn are associated with LRRK2-induced pathological mechanisms is still unknown. It has been reported that LRRK2 kinase activity regulates MAPK cascades [53, 54]. LRRK2-mediated stress response requires the activity of the MAPK kinase (MKK) 6 and p38 pathway, although the MAPK cascade contribution to pathogenesis remains unclear [53]. LRRK2 binds and phosphorylates MKK 3, 6 and 7 [53, 54]. MKK3/6 and MKK7 are upstream effectors of signaling proteins p38 and JNKs, respectively [55], indicating that the MAPK pathway is downstream of LRRK2 kinase activity, possibly leading to LRRK2-associated PD.

In the present study, we investigated the roles of LRRK2 and its kinase activity in Mn-induced toxicity in human microglial cells (HMC3), as well as RAW 264.7 macrophage-like cells. To clearly delineate the role of LRRK2 in Mn toxicity, we utilized LRRK2 knockout (KO) RAW 264.7 cells. Our results showed that Mn increased LRRK2 kinase activity and expression, while LRRK2 deletion and inhibition of LRRK2 kinase activity afforded protection against Mn-induced toxicity in human microglia as well as macrophages.

## Materials and methods

### Materials

Manganese (II) chloride (MnCl_2_), Dimethyl sulfoxide (DMSO), 3,4,5-dimethyl thiazol-2,5-diphenyl tetrazolium bromide (MTT) and GSK2578215A (GSK) were purchased from Sigma-Aldrich (St. Louis, MO). MLi-2 was purchased from R&D Systems (Minneapolis, MN). PD 98059 and SB 203580 were purchased from Tocris (Littleton, CO). All cell culture media, including trypsin-EDTA, Minimum Essential Media (MEM), Dulbecco’s Modified Eagle Medium (DMEM) and Opti-MEM, were obtained from Gibco (Carlsbad, CA). The chloromethyl derivative of 2',7'-dichlorodihydrofluorescein diacetate (CM-H_2_DCFDA), an ROS molecular probe, was purchased from Invitrogen (Carlsbad, CA). Antibodies for extracellular signal-regulated kinase (ERK, sc-514302), phospho-ERK (sc-136521), c-Jun N-terminal kinase (JNK, sc-7345), phospho-JNK (sc-6254), Bcl-2-associated X (Bax, sc-7480), death domain-associated protein (Daxx, sc-8043) and β-actin (sc-47778) were obtained from Santa Cruz Biotechnology (Santa Cruz, CA). Antibodies for phospho-p38 (4511S) and p38 (9212) were from Cell Signaling Technology (Danvers, MA). Antibodies for phospho-LRRK2 (S1292, ab203181), rabbit anti-mouse IgG (ab6728) and goat anti-rabbit IgG (ab97051) were from Abcam (Cambridge, MA). LRRK2 antibody (NB300-268) was from Novus Biologicals (Centennial, CO). Annexin-V staining buffer (420201), Annexin-V binding buffer (422201) and FITC-Annexin-V (640906) were purchased from Biolegend (San Diego, CA). Propidium iodide (PI, P4170) was obtained from Sigma-Aldrich. The TNF-α standard tetramethylbenzidine (TMB) enzyme-linked immunosorbent assay (ELISA) development kit for murine (900-T54) and human (900-T25) cells were acquired from Peprotech (Rocky Hill, NJ). All chemicals were prepared in phosphate-buffered saline (PBS), double-distilled H_2_O or DMSO and diluted to working concentrations in Opti-MEM prior to use.

### Cell culture

LRRK2-wild-type (WT, SC-6003) and LRRK2-knockout (KO, SC-6004) RAW 264.7 macrophage and human embryonic microglia clone 3 (HMC3, CRL-3304) cell lines were obtained from American Type Culture Collection (ATCC, Manassas, VA). RAW 267.4 macrophage cultures were maintained in DMEM supplemented with 10% fetal bovine serum, 100 U/mL of penicillin and 100 μg/mL of streptomycin. HMC3 cells were maintained in MEM, with all other conditions identical to RAW 267.4 cells. Cells were dissociated using 0.25% trypsin-EDTA (Gibco), then plated in 96-well plates for ROS/MTT assays or 6-well plates for flow cytometry, mRNA or protein analysis. All cells were maintained at 37°C in a 95% air, 5% CO_2_ incubator.

### Western blot analysis

After treatment with the designated compounds, RAW 264.7 and HMC3 cells were washed with ice-cold PBS. Cells were lysed by adding a radioimmunoprecipitation assay (RIPA) buffer and a protease inhibitor cocktail and then harvested. The protein concentration of the lysate was determined by bicinchoninic acid (BCA) assay. Thirty (30) μg or 200 μg (for p-LRRK2 and total LRRK2) of protein per sample was mixed with 4X Laemmli buffer and 5% β-mercaptoethanol in a 3:1 ratio, then heated at 95°C for 5 min. The samples were run on 10% or 6% (for p-LRRK2 and total LRRK2) SDS-PAGE gels and transferred to a nitrocellulose membrane for Western blot analysis. The primary antibodies were used at a 1:1000 dilution and secondary antibodies were used at a 1:5000 dilution. All blots were developed using a Pierce chemiluminescence detection kit (Rockford, IL), followed by blot imaging and quantification with the Molecular Imager ChemiDoc XRS+ System (Bio-Rad).

### Cell viability assay

RAW 264.7 and HMC3 cells (2×10^4^/well) were grown in 96-well plates, then exposed Mn (250 μM) for 24 h. For some experiments, inhibitors of LRRK2 and MAPK were pretreated for 90 min prior to Mn exposure. After incubation for the designated time period, cells were washed twice with ice-cold PBS. Ten μl of 3-(4,5-dimethylthiazol-2-yl)-2,5-diphenyltetrazolium bromide (MTT) (5 mg/ml in PBS) was added and cells were incubated for 3 h at 37°C. After incubation, 100 μl of 0.1 N hydrochloric acid (HCl) was added and the absorbance of the converted dye was measured at a wavelength of 570 nm using a microplate reader.

### Flow cytometry

Cellular apoptosis was determined by an annexin V-fluorescein isothiocyanate (FITC)/propidium iodide (PI) staining using fluorescence-activated cell sorting (FACS). RAW 264.7 and HMC3 cells were pre-treated with GSK (1 μM), an LRRK2 kinase activity inhibitor, for 1.5 h. Mn (250 μM) was subsequently added and the cells were incubated at 12-h and 24-h time points. Briefly, cells were dissociated via trypsinization, washed twice and re-suspended in a binding buffer. One hundred (100) μl aliquots containing 1.0 x 10^6^ cells per sample were used for the experiment. Following resuspension, cells were stained sequentially with Annexin V-FITC (5 μg/μl, Biolegend) and PI (5 μg/μl, Sigma Aldrich) according to the manufacturer’s instructions. After incubation for 15 min, 400 μl of binding buffer was added. Apoptotic cells were analyzed by a BD Facscalibur 2.0 flow cytometer (BD Biosciences, San Jose, CA) using Flowing Software V.2 (Turku BioImaging, Turku, Finland).

### Oxidative stress

Generation of ROS as an indicator of oxidative stress was measured using the Life Technologies CM-H_2_DCFDA ROS molecular probe, in accordance with manufacturer protocols. Briefly, RAW 264.7 and HMC3 cells were washed with PBS and treated with Mn (250 μM, 3 h) at 37°C. To determine the roles of LRRK2 kinase activity and MAPK signaling on Mn-induced oxidative stress, the pharmacological inhibitors such as GSK, MLi-2, PD 98059 and SB 203580 were pretreated for 90 min prior to Mn exposure. Cells were washed and 2.5 μM of CM-H_2_DCFDA was added for 10 min. Endpoint fluorescence was determined at an excitation/emission wavelength of 485/527 nm using the Spectramax i3x Multi-mode microplate reader (Molecular Devices, San Jose, CA).

### ELISA

TNF-α release from RAW 264.7 and HMC3 cells was determined using murine and human TNF-α ELISA kits (Peprotech) according to the manufacturer’s instructions. Briefly, the media (1 mL/well) were collected from the sample after exposure to Mn for the designated periods. ELISA was then performed and the optical density of each well measured. The concentration of secreted TNF-α was determined using a multi-mode microplate reader (Molecular Devices) set to 450 nm, with wavelength correction set at 620 nm.

### Real-time quantitative PCR (qPCR) analysis

RAW 264.7 and HMC3 cells were harvested after treatment with the designated compounds (3 samples/group). Total RNA was extracted from samples using the RNeasy Mini Kit (Qiagen, Valencia, CA) and 2 μg of purified RNA was transcribed to cDNA with a high-capacity cDNA reverse transcription kit (Applied Biosystems, Foster City, CA). Real-time qPCR was performed for TNF-α using the CFX96 real-time PCR detection system (Bio-Rad). The reaction mixture contained 1 μl of each cDNA template, 0.4 μM of primers and iQ SYBR Green Supermix (Bio-Rad, Hercules, CA). The total reaction volume was 25 μL. The following primers were used: human LRRK2, 5’-TGT TGT GGA AGT GTG GGA TAA-3’ (forward) and 5’- CAT TTT TAA GGC TTC CTA GCT G-3’ (reverse); human GAPDH, 5’-AAT GGG CAG CCG TTA GGA AA-3’ (forward) and 5’-GCG CCC AAT ACG ACC AAA TC-3’ (reverse); mouse LRRK2, 5’-GTC ATG GCA CAG ATC TTG ACA GTG AAG GTG GA-3’ (forward) and 5’-GTC TAA GAC TTC AGA GCC TAC CAG ACA GTA TGC TT-3’ (reverse); mouse TNF-α, 5’-GGT CCC CAA AGG GAT GAG AAG TTC-3’ (forward), 5’-CCA CTT GGT GGT TTG CTA CGA CG-3’ (reverse); mouse GAPDH, 5’-CTC ATG ACC ACA GTC CAT GC-3’ (forward) and 5’-CAC ATT GGG GGT AGG AAC AC-3’ (reverse). The qPCR parameters were set for 1 cycle at 95°C for 10 min, 40 cycles at 95°C for 15 s and 60°C for 1 min. GAPDH was utilized as an internal control. Following PCR, mRNA levels of TNF-α were analyzed using the Bio-Rad CFX Manager version 3.1.

### Statistical analysis

All data were expressed as the mean ± standard error of the mean (SEM). Multiple comparisons analyses were performed using one-way analysis of variance (ANOVA), followed by Tukey’s post hoc tests using the GraphPad Prism Software version 6.0 (San Diego, CA). A *p*-value of less than 0.05 (*p* < 0.05) was considered statistically significant.

## Results

### Mn increases LRRK2 kinase activity and expression

**Fig 1:**
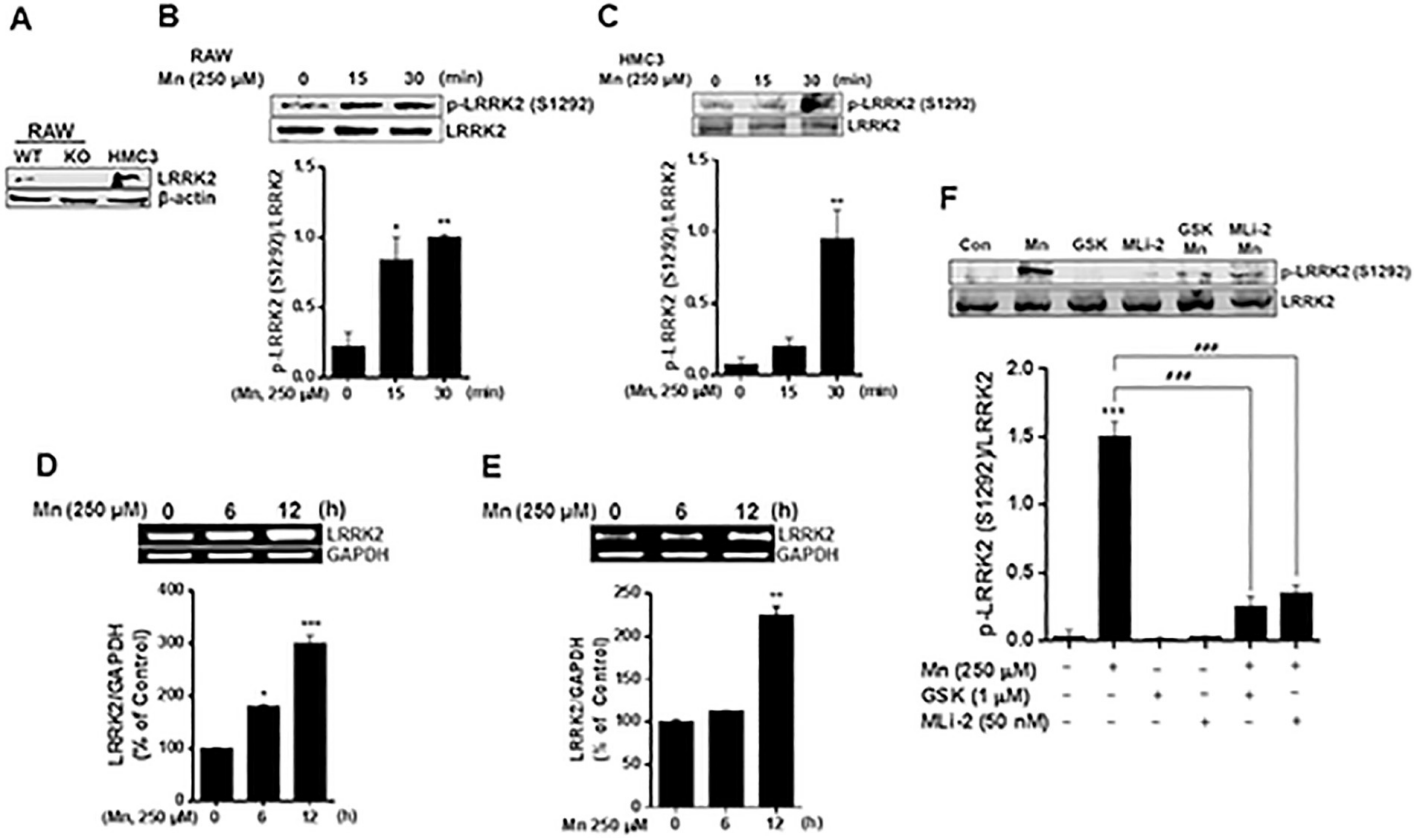
Mn increases LRRK2 kinase activity and expression in RAW 264.7 and HMC3 cells. (**A**) Two-hundred (200) μg of protein were collected from cell lysates, followed by western blot analysis to determine the presence of LRRK2 in LRRK2 WT, KO RAW 264.7 and HMC3 cells. β-actin was used as a loading control. (**B,C**) LRRK2 WT or HMC3 cells were treated with Mn (250 μM) for the designated times, followed by protein extraction and western blot analysis as described in the Methods section. Protein levels of LRRK2 and phosphorylated LRRK2 (S1292) in LRRK2 WT RAW 264.7 (B) and HMC3 (C) cells were quantified. (**D,E**) Effect of Mn on LRRK2 mRNA levels in LRRK2 WT RAW 264.7 (D) and HMC3 (E) cells were assessed as described in the Methods section. GAPDH was used as a loading control. (**F**) After pre-treatment with LRRK2 inhibitors GSK (1 μM) and MLi-2 (50 nM) for 90 min, RAW 264.7 cells were exposed to Mn (250 μM) for 20 min, followed by western blot analysis to detect phosphorylation of LRRK2 (S1292). ^###^, *p* < 0.001; *, *p* < 0.05; **, *p* < 0.01; ***, *p* < 0.001 compared to the control (one-way ANOVA followed by Tukey’s post hoc test; n = 3). The data shown are representative of 3 independent experiments.

To determine the potential role of LRRK2 in Mn toxicity, we tested if LRRK2 modulates Mn-induced oxidative stress and inflammation using murine macrophage LRRK2 WT, LRRK2 KO RAW 264.7 cells, and human microglia HMC3 cells. First, we confirmed the expression of LRRK2 in these cell lines, as well as the deletion of LRRK2 in LRRK2 KO RAW cells. As shown in Fig 1A, LRRK2 WT and HMC3 cells showed LRRK2 protein expression, while LRRK2 KO cells did not express LRRK2. Next, we tested if Mn activates LRRK2 kinase activity by assessing the phosphorylation of LRRK2 at serine 1292 (S1292) as an indicator of LRRK2 kinase activity [36, 56]. The results revealed that Mn increased phosphorylation of LRRK2 S1292 after a short period of Mn exposure in both RAW 264.7 (Fig 1B) and HMC3 cells (Fig 1C). As a recent study reported that Mn increased LRRK2 expression in BV2 microglia cells and mouse brains after prolonged Mn exposure [6], we also tested if Mn modulates LRRK2 expression in both RAW 264.7 (Fig 1D) and HMC3 cells (Fig 1E) after 12 h of Mn (250 μM) exposure.

Given that Mn activates LRRK2 expression and kinase activity, we confirmed that Mn-induced activation of LRRK2 is blocked by the inhibition of LRRK2 kinase activity using LRRK2 inhibitors GSK and MLi-2. The results showed that Mn-induced phosphorylation of LRRK2 at S1292 was inhibited by GSK and MLi-2 in RAW 264.7 cells (Fig 1F).

### Deletion of LRRK2 attenuates Mn-induced apoptosis and cell death in macrophage RAW 264.7 cells

**Fig 2:**
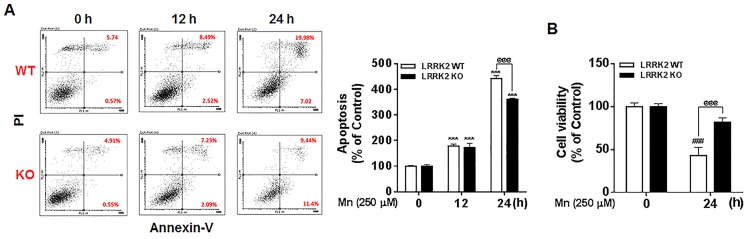
Deletion of LRRK2 attenuates Mn-induced apoptosis and cell death in RAW 264.7 cells. (**A**) LRRK2 WT or KO RAW 264.7 cells were treated with Mn (250 μM) for the designated times, followed by flow cytometry analysis to determine Mn-induced apoptosis. Both early and late apoptotic cells (Q2 and Q3) were measured. (**B**) At the end of Mn exposure, cell viability was determined by MTT assay. ^@@@^, *p* < 0.001; ^###^, *p* < 0.001; ***, *p* < 0.001 compared to the control (one-way ANOVA followed by Tukey’s post hoc test; n = 3, for apoptosis assay; n = 6, for MTT assay). The data shown are representative of 3 independent experiments.

Since we found that Mn increased LRRK2 kinase activity and expression, we tested if LRRK2 played a role in Mn-induced toxicity in human microglia, LRRK2 WT and KO RAW cells. These cells were exposed to Mn (250 μM) for 12 h and 24 h, followed by assessment of apoptosis and cell viability using flow cytometry and MTT assay, respectively. As shown in Fig 2A, the number of apoptotic cells increased after 12 h and 24 h of Mn exposure, while deletion of LRRK2 attenuated the toxic effects of Mn by significantly decreasing Mn-induced apoptotic cells—from 27.0% for early and late apoptosis to 20.8% for 24 h of Mn exposure. Mn exposure for 12 h resulted in no difference in the number of apoptotic LRRK2 KO cells as compared to LRRK2 WT cells. Late apoptotic signals were significantly attenuated in the absence of LRRK2, decreasing from 20.0% to 9.4% (Fig 2A). Mn also significantly decreased cell viability in LRRK2 WT cells after 24 h of Mn (250 μM) exposure; however, Mn-induced cell death was significantly attenuated in LRRK2 KO cells as compared to Mn-induced LRRK2 WT cells (Fig 2B).

### Inhibition of LRRK2 kinase activity attenuates Mn-induced apoptosis and cell death in macrophages and human microglia

**Fig 3:**
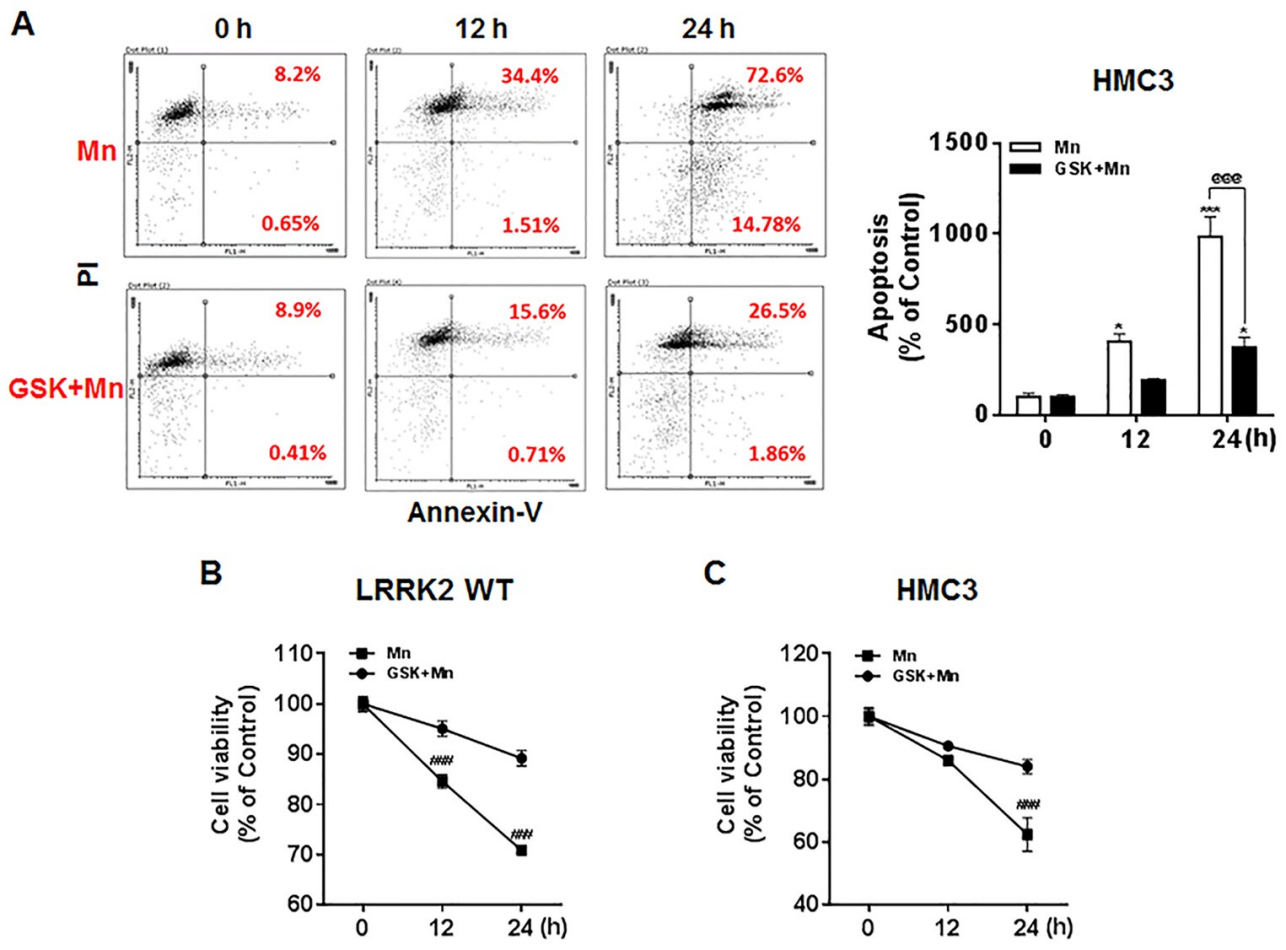
Inhibition of LRRK2 kinase activity attenuates Mn-induced apoptosis. (**A**) After pre-treatment with GSK (1 μM) for 90 min, cells (HMC3) were exposed to Mn (250 μM) for the designated time periods, followed by annexin V and PI staining and flow cytometry analysis to determine apoptosis. Early and late apoptotic cells (Q2 and Q3) were analyzed. (**B,C**) After pre-treatment with GSK (1 μM) for 90 min, cells (LRRK2 WT RAW 264.7 and HMC3) were exposed to Mn for designated time periods, followed by the MTT assay to determine cell viability, as described in the Methods section, (^@@@^, *p* < 0.001; *, *p* < 0.05; ***, *p* < 0.001 compared to the control (one-way ANOVA followed by Tukey’s post hoc test; n = 3, apoptosis assay; n = 6, MTT assay). The data shown are representative of 3 independent experiments.

Given that LRRK2 deletion induced attenuation of Mn toxicity, we next determined whether the effect of LRRK2 is associated with its kinase activity in Mn-induced apoptosis. As both MLi-2 and GSK inhibited Mn-induced activation of LRRK2, we used GSK as a representative to investigate whether LRRK2 kinase activity plays a role in Mn-induced toxicity in both RAW 264.7 and HMC3 cells. The results showed that inhibition of LRRK2 kinase activity attenuated Mn-induced apoptosis in human microglia HMC3 cells (Fig 3A). Moreover, the MTT assay showed that inhibition of LRRK2 kinase activity with GSK significantly attenuated Mn-induced cell death in both RAW 264.7 and HMC3 cells (Fig 3B and 3C). These results indicate that LRRK2 kinase activity plays an important role in Mn-induced apoptosis and cell viability in immune cells.

### Deletion of LRRK2 attenuates the Mn-induced pro-apoptotic protein Bax, but not Daxx in RAW 264.7 cells

**Fig 4:**
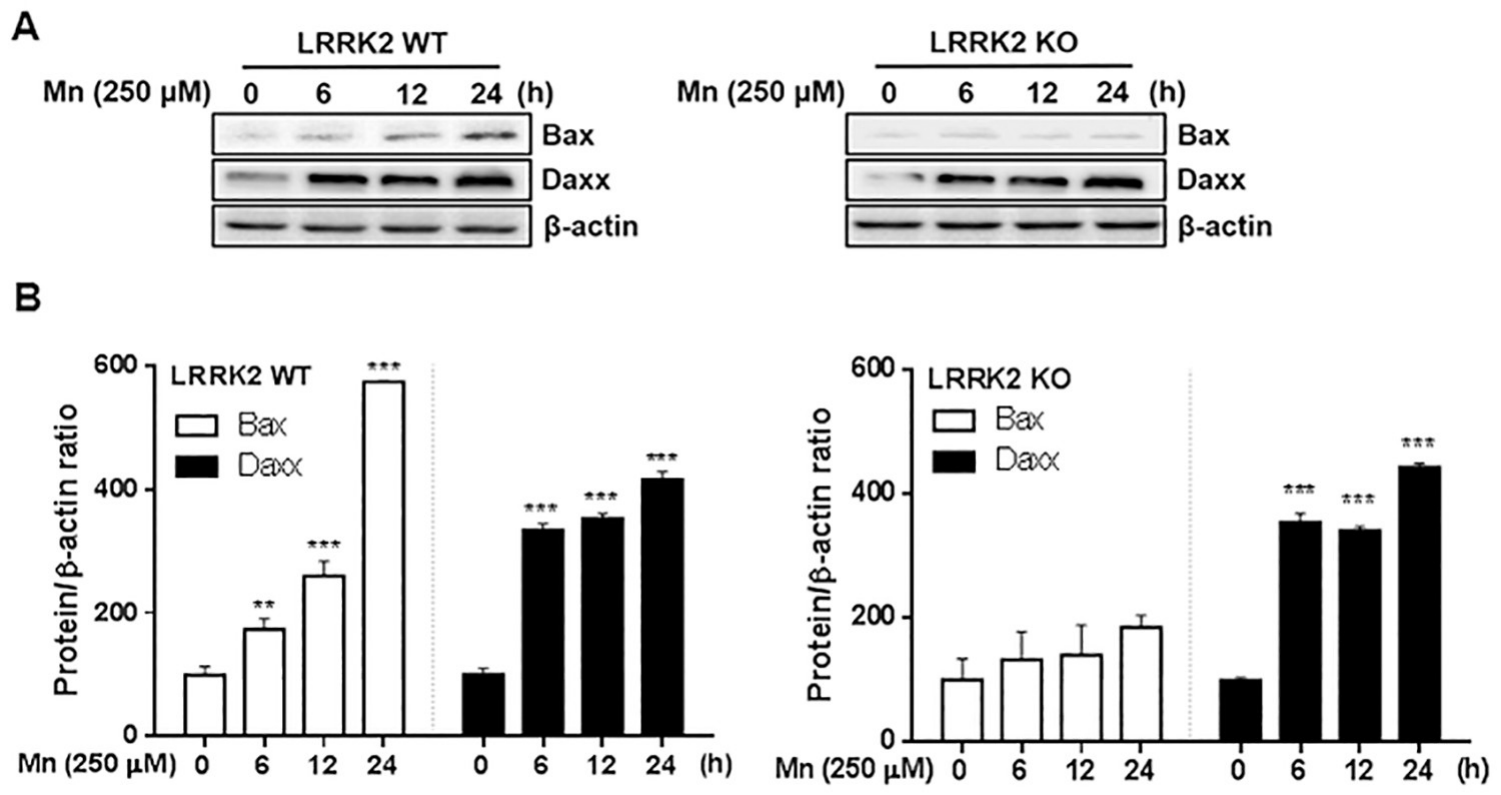
LRRK2 deletion attenuates Mn-induced pro-apoptotic protein levels. (**A**) After cells (LRRK2 WT and LRRK2 KO) were exposed to Mn (250 μM) for up to 24 h, total protein was extracted and followed by western blot analysis to determine protein levels of Bax and Daxx. β-actin was used as a loading control. (**B**) The expression levels of Bax and Daxx modulated by Mn were quantified relative to its control levels in LRRK2 WT and KO cells. **, *p* < 0.01; ***, *p* < 0.001 (one-way ANOVA followed by Tukey’s post hoc test; n = 3). The data shown are representative of 3 independent experiments.

We subsequently examined if the apoptotic signaling proteins Bax and Daxx were involved in Mn-induced toxicity via LRRK2 kinase activity. As shown in Fig 4A and 4B, Mn significantly increased the levels of the pro-apoptotic protein Bax in LRRK2 WT RAW 264.7 cells. However, Mn did not increase Bax levels in LRRK2 KO cells (Fig 4A and 4B), indicating that LRRK2 is likely involved in Mn-induced Bax expression. On the other hand, Mn increased Daxx protein levels independent of LRRK2 in both LRRK2 WT and LRRK2 KO cells.

### Inhibition of LRRK2 kinase attenuates Mn-induced Bax in RAW 264.7 cells, but Bax and Daxx in microglia

**Fig 5:**
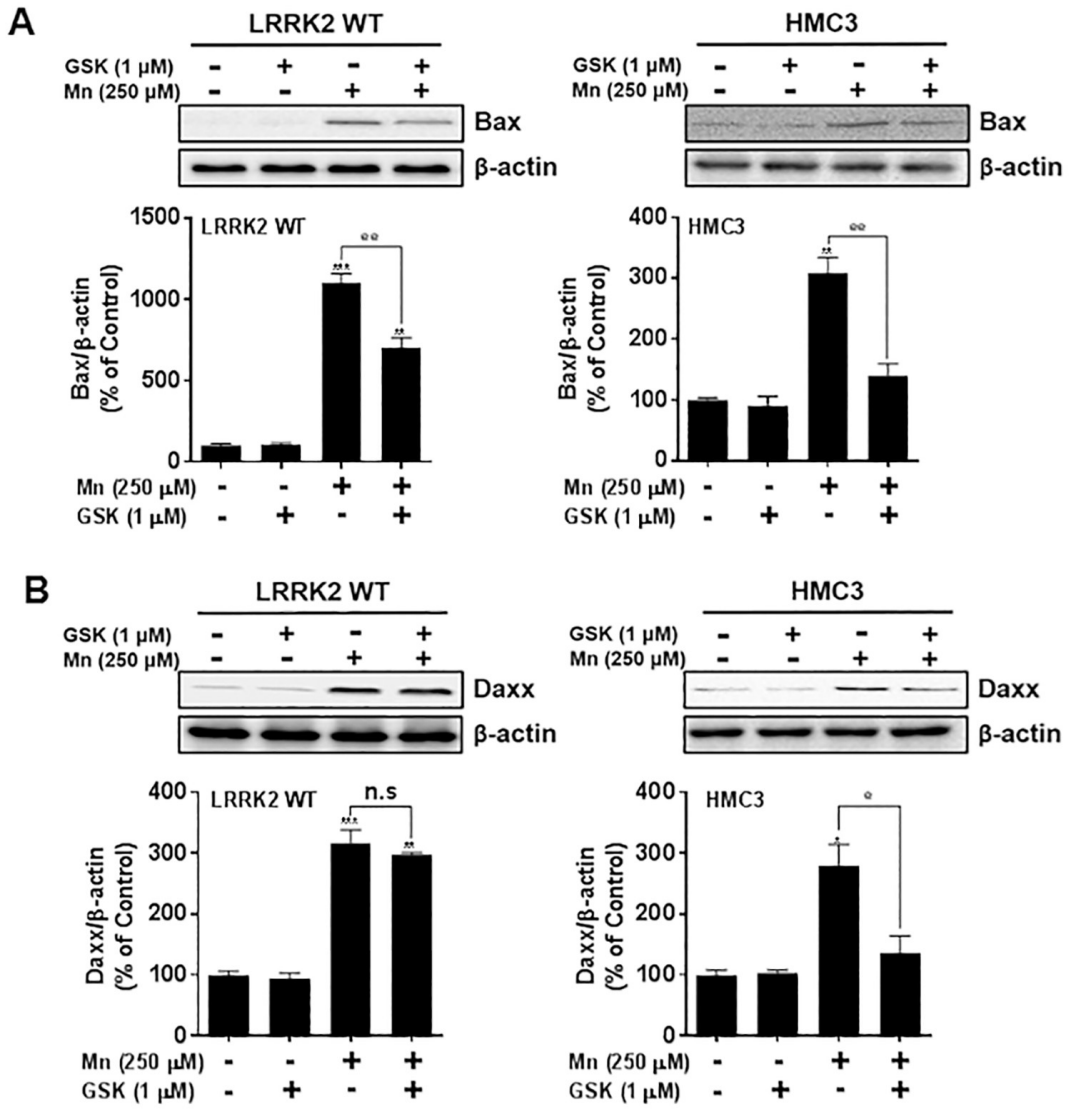
Inhibition of LRRK2 kinase activity attenuates Mn-induced pro-apoptotic protein levels. (**A,B**) After pre-treatment with GSK (1 μM) for 90 min, RAW 264.7 and HMC3 cells were exposed to Mn (250 μM) for 12 h, followed by extraction of protein and western blot analysis to determine protein levels of Bax (**A**) and Daxx (**B**). β-actin was used as a loading control. ^@^, *p* < 0.05; ^@@^, *p* < 0.01; *, *p* < 0.05; **, *p* < 0.01; ***, *p* < 0.001 (one-way ANOVA followed by Tukey’s post hoc test; n = 3). The data shown are representative of 3 independent experiments.

Next, we tested if LRRK2 kinase activity plays a role in Mn-induced Bax and Daxx expression. Inhibition of LRRK2 kinase with GSK attenuated the Mn-induced Bax expression in both RAW 264.7 and human microglia HMC3 cells (Fig 5A). Interestingly, inhibition of LRRK2 kinase attenuated Mn-induced Daxx expression in human microglia HMC3, but not in LRRK2 WT RAW 264.7 cells (Fig 5B).

### Deletion or inhibition of LRRK2 attenuates Mn-induced oxidative stress in RAW 264.7 and human microglia

**Fig 6:**
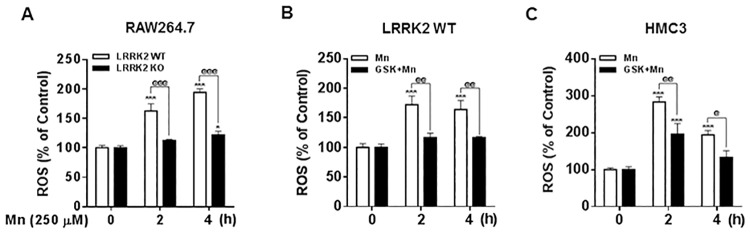
Inhibition of LRRK2 attenuates Mn-induced oxidative stress. (**A**) After cells (LRRK2 WT and KO RAW 264.7) were exposed to Mn (250 μM) for the designated time periods, ROS were measured by fluorometer using DCF-fluorescence reagent to determine oxidative stress as described in the Methods section. (**B,C**) After pre-treatment with GSK (1 μM) for 90 min, LRRK2 WT RAW 264.7 (B) and HMC3 (C) cells were exposed to Mn (250 μM), followed by ROS measurement by a fluorometer. ^#^, *p* < 0.05; ^##^, *p* < 0.01; ^###^, *p* < 0.001 compared to the control (one-way ANOVA followed by Tukey’s post hoc test; n = 6). The data shown are representative of 3 independent experiments.

It is well established that Mn induces oxidative stress in many different cell types [10], and that oxidative stress is an important indicator of mitochondrial damage and cell death in several neurodegenerative models including manganism and PD. Since LRRK2 deletion and kinase activity inhibition attenuated Mn-induced apoptosis and cell death, next, we examined whether LRRK2 may play a role in Mn-induced oxidative stress. As shown in Fig 6A, Mn (250 μM, 1h) exposure in LRRK2 WT RAW 264.7 cells produced ROS determined by DCF-fluorescence assay, while LRRK2 deletion significantly blocked Mn-induced ROS, indicating that LRRK2 plays a role in Mn-induced oxidative stress. We also examined if LRRK2 kinase activity plays a role in Mn-induced ROS production. The results showed that inhibition of LRRK2 kinase activity attenuated Mn-induced ROS in both RAW 264.7 (Fig 6B) and human microglia HMC3 cells (Fig 6C). Considering these findings, LRRK2 kinase activity is posited to play a role in Mn-induced oxidative stress.

### Deletion or inhibition of LRRK2 attenuates Mn-induced TNF-α in RAW 264.7 and human microglia

**Fig 7:**
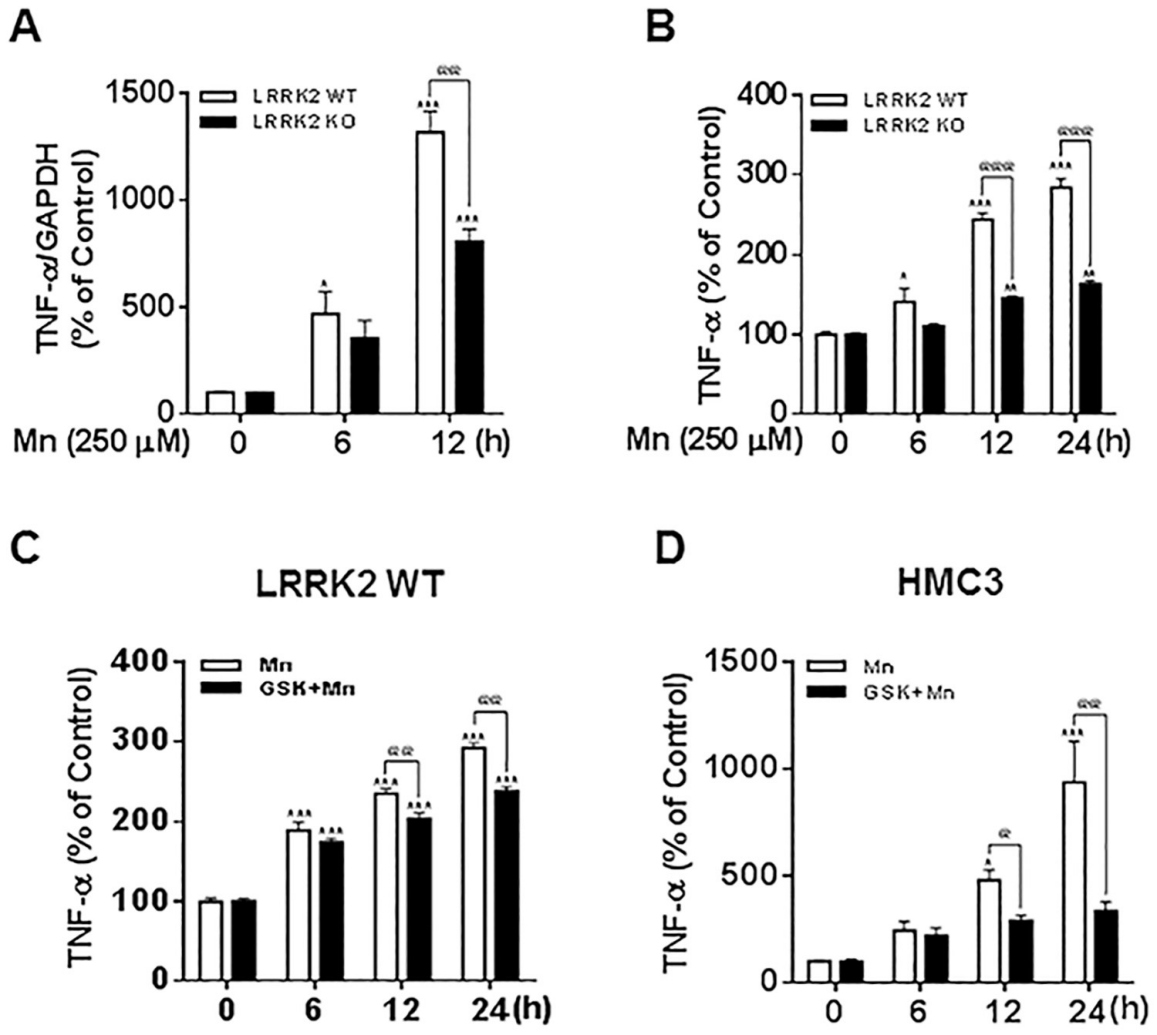
Inhibition of LRRK2 attenuates Mn-induced TNF-α production. (**A,B**) After RAW 264.7 cells were exposed to Mn (250 μM), TNF-α mRNA levels and (A) TNF-α secretion (B) were measured by qPCR (A) and ELISA (B) as described in the Methods section. (**C,D**) After pre-treatment with GSK (1 μM) for 90 min, RAW 264.7 (C) and HMC3 (D) cells were exposed to Mn (250 μM), followed by ELISA to determine TNF-α secretion. ^@@^, *p* < 0.01; ^@@@^, *p* < 0.001; *, *p* < 0.05; **, *p* < 0.01; ***, *p* < 0.001 compared to the control (one-way ANOVA followed by Tukey’s post hoc test; n = 3). The data shown are representative of 3 independent experiments.

Since previous studies have shown that Mn induces inflammation in microglia [23], we sought to find if deleting or inhibiting LRRK2 kinase activity also attenuates Mn-induced inflammation. As shown in Fig 7A, Mn increased mRNA levels of TNF-α at 6 h and 12 h. The deletion of LRRK2 significantly attenuated Mn-induced mRNA levels of TNF-α at 12 h in RAW 264.7 cells. Mn-induced secretion of TNF-α as determined by ELISA was also significantly attenuated in LRRK2 KO cells (Fig 7B). We further tested if the kinase activity of LRRK2 is involved in Mn-induced TNF-α production. The results showed that inhibition of LRRK2 kinase activity with GSK attenuated Mn-induced TNF-α secretion in RAW 264.7 macrophages (Fig 7C) as well as in human microglia HMC3 cells (Fig 7D). These results indicate that LRRK2 kinase activity is likely involved in Mn-induced pro-inflammatory cytokine TNF-α production in microglia.

### Deletion of LRRK2 attenuates Mn-induced phosphorylation of MAPK p38 and ERK in RAW 264.7 cells

**Fig 8:**
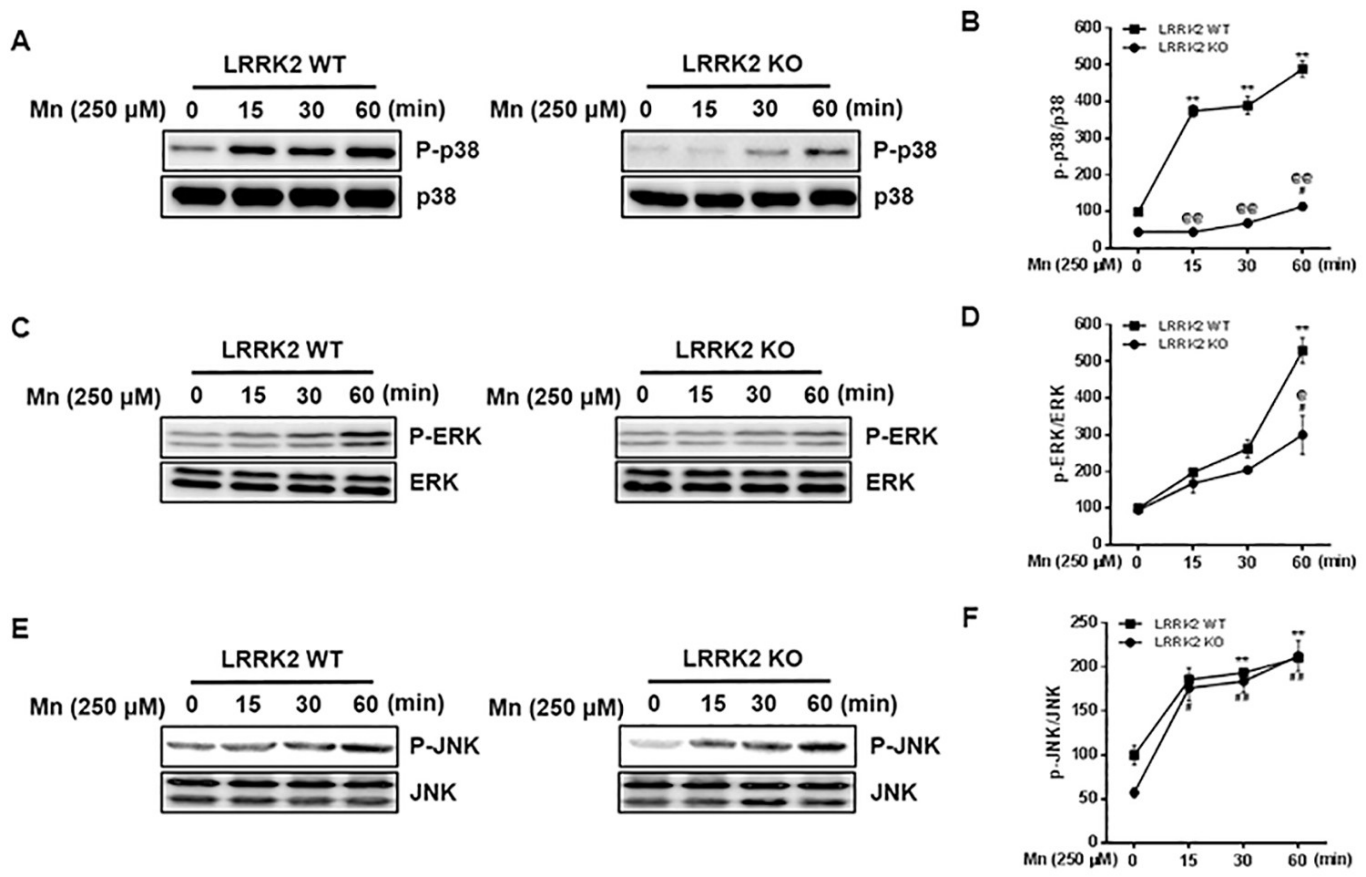
LRRK2 mediates Mn-induced MAPK p38 and ERK, but not JNK signaling in RAW 264.7 cells. (**A,C,E**) After LRRK2 WT and KO cells were exposed to Mn (250 μM) for up to 60 min, the protein was extracted, followed by western blot analysis to detect phosphorylation of signaling proteins as described in the Methods section. (**B,D,F**) The levels of total and phosphorylated MAPK p38 (B), ERK (D) and JNK (F) were quantified. **, *p* < 0.01; ***, *p* < 0.001 compared to the WT control. ^##^, *p* < 0.01; ^###^, *p* < 0.001 compared to the KO control. ^@@^, *p* < 0.01; ^@@@^, *p* < 0.001 compared between WT and KO at the same time points. (one-way ANOVA followed by Tukey’s post hoc test; n = 3). The data shown are representative of 3 independent experiments.

After observing that LRRK2 played a role in Mn-induced oxidative stress, inflammation and apoptosis, we then investigated if the MAPK signaling pathway plays a role in Mn-induced toxicity via LRRK2 kinase activation. MAPK signaling pathways are known to be involved in various cellular processes and stress response [57]. Previous studies have reported that LRRK2 phosphorylates several signaling proteins MAPK p38, ERK and JNK in association with cell proliferation and apoptosis [58, 59]. The results showed that Mn induced phosphorylation of MAPK p38, ERK and JNK in RAW 264.7 cells, but Mn-induced activation of signaling proteins MAPK p38 and ERK were significantly attenuated in LRRK2 KO cells (Fig 8A–8D). Interestingly, LRRK2 deletion did not attenuate the Mn-induced activation of JNK signaling (Fig 8E and 8F).

### LRRK2-MAPK signaling is involved in Mn-induced toxicity in immune cells

**Fig 9:**
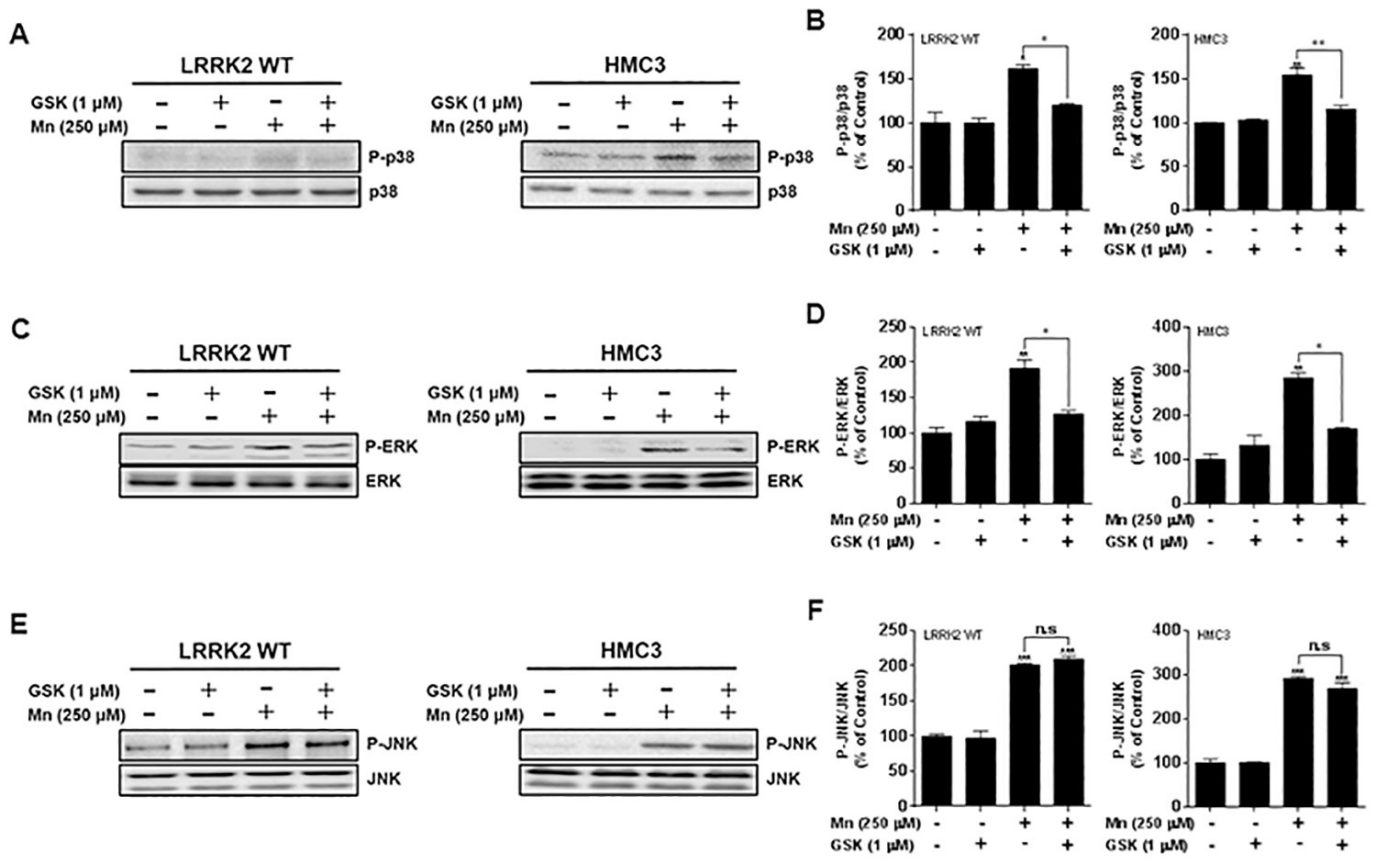
Inhibition of LRRK2 kinase activity attenuates Mn-induced MAPK p38 and ERK, but not JNK in RAW 264.7 and HMC3 cells. (**A,C,E**) After pre-treatment with GSK (1 μM) for 90 min, cells were exposed to Mn (250 μM) for 30 min, followed by total protein extraction and western blot analysis as described in the Methods section. (**B,D,F**) The levels of total and phosphorylated p38 (B), ERK (D) and JNK (F) were quantified. ^@^, *p* < 0.05; ^@@^, *p* < 0.01; *, *p* < 0.05; **, *p* < 0.01; ***, *p* < 0.001 compared to the control (one-way ANOVA followed by Tukey’s post hoc test; n = 3). The data shown are representative of 3 independent experiments.

Given that LRRK2 is involved in Mn-induced activation of MAPK signaling proteins, we sought to determine whether LRRK2 kinase activity plays a role in the effects of Mn on MAPK signaling. The results showed that Mn increased the activation of MAPK p38, ERK and JNK in both LRRK2 WT RAW 264.7 cells and human microglia, whereas inhibition of LRRK2 kinase activity with GSK significantly attenuated Mn-induced phosphorylation of MAPK p38 and ERK in both macrophages and microglia (Fig 9A–9D). On the other hand, GSK inhibition of LRRK2 kinase activity did not block Mn-induced JNK phosphorylation in both LRRK2 WT RAW 264.7 cells and microglia (Fig 9E and 9F), indicating that LRRK2 is not involved in Mn-induced activation of JNK signaling in these cell types.

**Fig 10:**
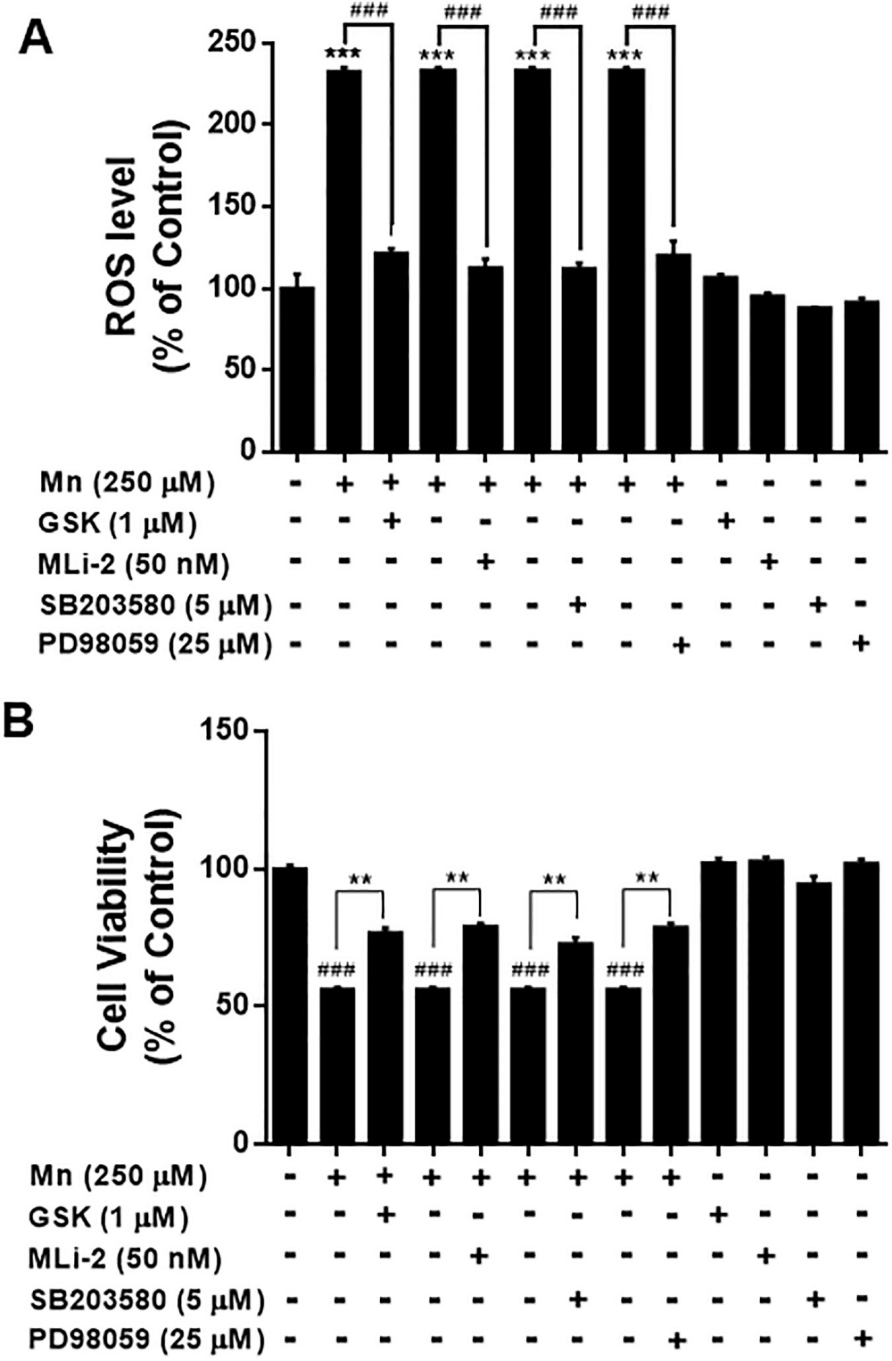
Inhibition of p38 and ERK attenuates Mn-induced oxidative stress and cell death. (**A,B**) After pre-treatment with LRRK2 inhibitors GSK (1 μM) and MLi-2 (50 nM), p38 inhibitor SB 203580, or ERK inhibitor PD 98059 for 90 min, cells were exposed to Mn (250 μM) for the designated time periods, followed by ROS and MTT assays as described in the Methods section, (^###^, *p* < 0.001; **, *p* < 0.01; ***, *p* < 0.001 compared to the control (one-way ANOVA followed by Tukey’s post hoc test; n = 6). The data shown are representative of 3 independent experiments.

LRRK2 is known to be upstream of MAPK kinases, phosphorylating MKK 3, 6 and 7 [53, 54], which could suggest that LRRK2-MAPK signaling might be involved in Mn toxicity. Since Mn activated both MAPK signaling and LRRK2 (Figs 1 and 9) and the inhibition of LRRK2 abolished Mn-induced MAPK activation as well as Mn toxicity (Figs 3, 6, 7 and 9), we tested if inhibition of Mn-induced MAPK p38 and ERK activation also attenuates Mn toxicity via LRRK2. The results showed that inhibition of MAPK p38 and ERK signaling with SB 203580 and PD 98059, respectively, as well as inhibition of LRRK2 with GSK and MLi-2, attenuated Mn-induced oxidative stress (Fig 10A) and cytotoxicity (Fig 10B) in RAW 264.7 cells.

## Discussion

Our findings from the present study demonstrate that LRRK2 plays an important role in Mn-induced oxidative stress, inflammation and apoptosis in microglia. In addition, for the first time we report that Mn increases LRRK2 kinase activity in macrophages and microglia. We also found that LRRK2 kinase activity mediates Mn toxicity via the MAPK signaling pathway, as deletion of LRRK2 and inhibition of LRRK2 kinase activity abolished Mn-activated MAPK signaling. These results indicate that the LRRK2-MAPK signaling pathway plays an important role in Mn-induced toxicity in immune cells.

Studies on the involvement of LRRK2 in Mn-induced inflammation and toxicity are scarce, but it has recently been reported that microglial LRRK2 is involved in Mn-induced neuroinflammation and concomitant impairment of the dopaminergic system [6]. Their findings showed that LRRK2 is necessary for Mn-induced toxicity, particularly autophagy and inflammation in microglia in fact support and corroborate the results presented herein. Although it has been shown that Mn increased LRRK2 expression [6], whether Mn induces phosphorylation of LRRK2 was previously unknown. Notably, the present study reveals that Mn did activate LRRK2 kinase activity, which was indeed involved in Mn-induced toxicity in microglia. This LRRK2 kinase activation could be due to Mn’s direct binding to the LRRK2 kinase domain [32], potentially leading to pathogenic consequences [60]. Enhanced LRRK2 kinase activity is believed to contribute to LRRK2 G2019S mutant-induced PD pathogenesis, as this mutation is positioned in the kinase domain [61]. Mn also increased LRRK2 expression in both macrophages and microglia, indicating that Mn may enhance LRRK2 at the transcriptional level. Interestingly, another toxicant, rotenone, also increased LRRK2 expression in microglia [62].

Our findings reveal that LRRK2 plays a significant role in Mn-induced toxicity, including inflammation. Moreover, Mn-induced toxicity appears to be mediated by LRRK2 kinase, as LRRK2 deletion and GSK inhibition of LRRK2 abrogated Mn-induced toxic effects in microglia. This role of LRRK2 kinase activity in inflammation has been supported by findings that the LRRK2 G2019S mutation, known to increase LRRK2 kinase activity, exacerbated inflammation in G2019S LRRK2 transgenic rats [48]. Chronic exposure to elevated levels of Mn consistently induces the persistent activation of microglia, leading to the release of inflammatory molecules and ROS and resulting in neurodegeneration [11, 23, 24, 63]. Although astrocytes also significantly contribute Mn-associated neuronal injury and neurodegeneration, microglia play a major role in neuroinflammation associated with Mn toxicity [64]. Inflammation and oxidative stress are closely associated with the pathogenesis of many neurodegenerative disorders including AD, PD and multiple sclerosis [65]. Numerous studies have reported that LRRK2 is involved in neuroinflammation and inflammatory signaling [66, 67]. Microglial LRRK2 is linked to the inflammatory response associated with PD [68, 69]. In this context, LRRK2 is involved in Mn-induced neuroinflammation and microglial autophagy, as knockdown of LRRK2 with shRNA attenuated Mn effects in BV2 cells and a mouse model [6]. We also found that Mn increased the expression and secretion of the pro-inflammatory cytokine TNF-α in human microglia HMC3 cells. Notably, we found that not only LRRK2 deletion, but inhibition of LRRK2 kinase activity attenuated Mn-induced TNF-α production (Fig 7), suggesting that Mn may induce inflammation via LRRK2 kinase activity. As the inhibition of LRRK2 did not completely block Mn-induced TNF-α production, this indicates that Mn can induce inflammation in the absence of LRRK2 kinase activity, possibly through another mechanism.

Mn-induced oxidative stress appears to occur downstream of LRRK2 kinase activity, since LRRK2 inhibition and LRRK2 deletion ameliorated Mn-induced ROS production. Mn also increased phosphorylation at S1292 position of LRRK2 in human microglia (Fig 1C). Previous studies have reported the potential involvement of LRRK2 activation in oxidative stress; rotenone, a mitochondrial complex I inhibitor that is frequently used to generate PD experimental models, significantly increased oxidative stress with concomitant phosphorylation at S1292 of LRRK2 (an indicator of LRRK2 kinase activity) in microglia as well as in brains of the PD patient and a rat PD model [70]. Moreover, LRRK2 G2019S mutation is associated with increased ROS production, suggesting that oxidative stress is likely increased in the CNS of the individuals with PD-associated LRRK2 mutations [71]. Collectively, these findings indicate that LRRK2 kinase plays a role in Mn-induced oxidative stress.

Mn-induced apoptosis is closely associated with mitochondrial dysfunction and oxidative stress [72]. We found that Mn-induced LRRK2 activation increased the pro-apoptotic protein Bax. Bax increases the opening of the mitochondrial voltage-dependent anion channel, resulting in the loss of membrane potential and release of cytochrome c [73], thus leading to increased oxidative stress and activation of apoptotic signaling. Notably, Mn-induced levels of Daxx were unaffected by LRRK2 deletion or inhibition in macrophage cells, whereas inhibition of LRRK2 kinase attenuated Mn-induced Daxx in human microglia, suggesting that the role of LRRK2 kinase activity might be cell type-specific. Although many features of microglia, the resident brain macrophages, share those of peripheral macrophages, some toxicant-induced mechanisms may be differentially regulated. Daxx is involved in Fas-induced apoptosis via JNK signaling by activating the upstream JNK kinase, ASK1 [74–76]. It is likely that LRRK2 modulatory effects on Mn-induced Daxx may involve different cellular and molecular mechanisms. Further studies are required to determine the precise mechanisms of LRRK2 in Mn-induced inflammation and apoptosis in different cell types.

Our study finds that the MAPK signaling pathway is downstream of Mn-induced LRRK2 kinase activity, as part of the toxic mechanism of Mn. Corroborating these results, LRRK2 induced inflammation, with concomitant dysregulation of the p38 MAPK/Drosha pathway, was induced upon intracerebral hemorrhage in rats [15]. LRRK2 is also known to phosphorylate MKK3/6 and MKK4/7, the upstream kinases of MAPK, p38 and JNKs, respectively [53, 54]. Our findings show that MAPK p38 and ERK are involved in Mn-activated LRRK2 downstream activation. The discovery that LRRK2 kinase is involved in Mn-activated p38 is supported by other studies that demonstrated inhibition of LRRK2 attenuates human immunodeficiency virus 1 (HIV)-induced activation of LRRK2, p38 signaling and inflammation in primary microglia [77]. ERK, downstream of MKK1/2, is also activated via Mn-induced LRRK2 kinase activation, as GSK attenuated Mn-induced ERK activation. Pharmacological inhibition of LRRK2 and MAPK signaling proteins p38 and ERK ameliorated Mn-induced toxicity, indicating that LRRK2-MAPK signaling mediates Mn toxicity. Other studies also reported that activation of p38 and ERK are involved in LRRK2-G2019S-induced apoptosis and abnormal neurite growth, potentially implicating G2019S mutant-induced LRRK2 kinase activity in this process [41, 51, 78, 79]. It has also been reported that LRRK2 regulates p38 phosphorylation through a MKK-dependent pathway [54, 80, 81] or MKK—independent pathways [51]. It appears that in addition to Mn, other toxicants that activate MAPK signaling may also be influenced by LRRK2-MAPK signaling as we observed that the PD inducer MPP^+^ (1-methyl-4-phenylpyridinium), known to induce MAPK signaling and oxidative stress [82], increased ROS production, but inhibition of LRRK2 kinase with GSK and MLi-2 abolished MPP^+^-induced oxidative stress in RAW 264.7 cells (S1 Fig). On the other hand, LRRK2 G2019S mutant has been purported to activate the MKK4-JNK pathway and cause degeneration of SN dopaminergic neurons in a transgenic mouse model of PD [83]. However, our findings indicate that Mn-induced phosphorylation of JNK is not mediated by LRRK2, raising the possibility that Mn-induced JNK activation could be by some unknown mechanism. Nonetheless, these results indicate that Mn-induced activation of MAPK p38 and ERK signaling via LRRK2 kinase is significant for Mn-induced oxidative stress, inflammatory responses and toxicity.

Given the prominence of LRRK2 pathology in PD pathogenesis, LRRK2 may also play a role in other neurodegenerative diseases such as ischemia, traumatic brain injury and AD. It has been reported that LRRK2 could be promoting post-ischemic apoptotic cell death by modulating Tau phosphorylation in experimental cerebral ischemia [84]. Moreover, LRRK2 R1628P mutant increases the risk of AD, inducing a higher rate of apoptosis in mutant-expressing cells as compared to control cells [85]. At the cellular level, elevated LRRK2 kinase activity is associated with various pathological processes such as defects in protein synthesis and degradation, apoptosis and oxidative damage [86–88]. This indicates that LRRK2 plays a role in various neurodegenerative diseases, warranting further investigation to advance our understanding of LRRK2-related pathology.

**Fig 11:**
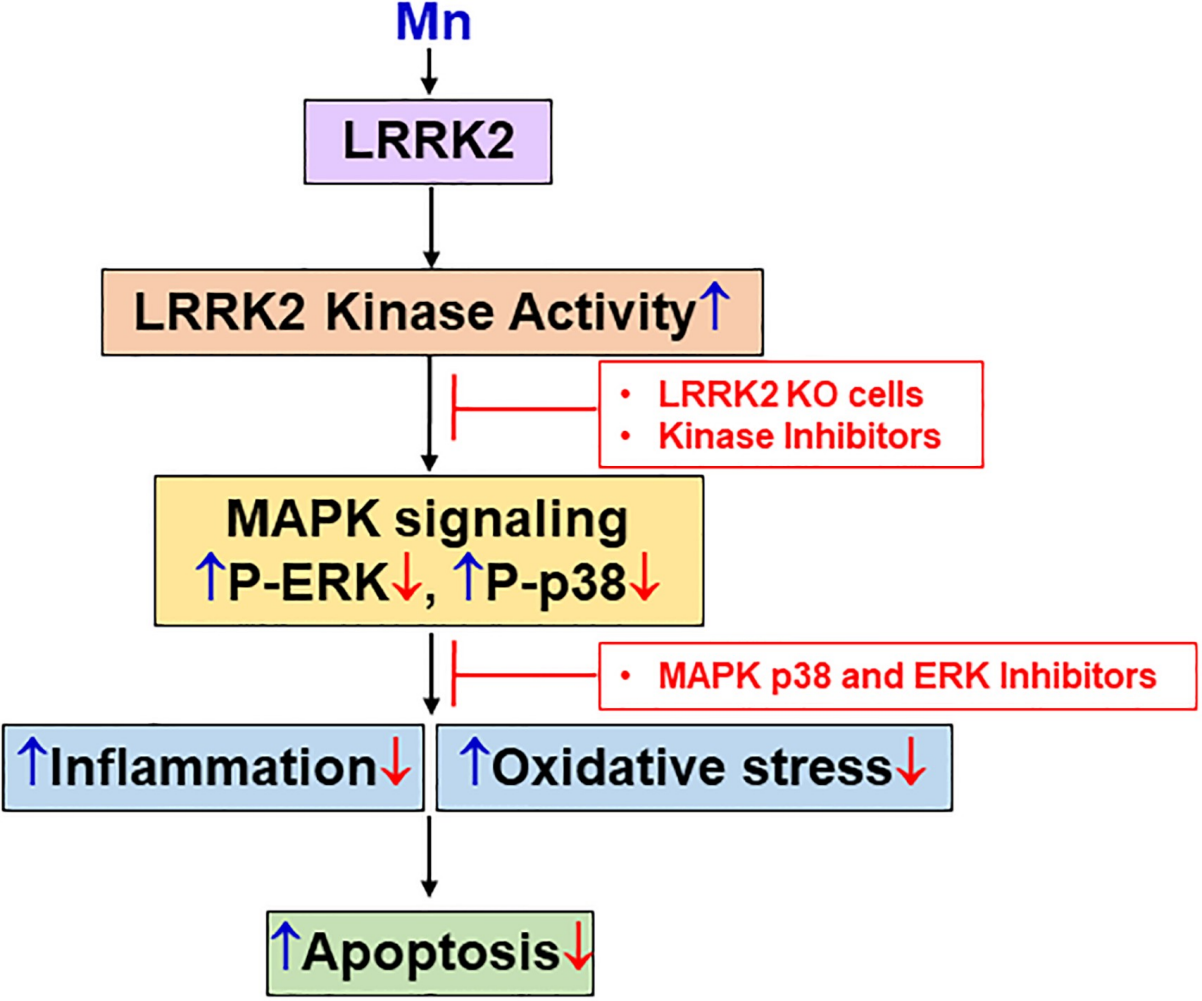
A proposed mechanism for the LRRK2-mediated Mn toxicity in microglia. Mn targets LRRK2 at the kinase domain to induce oxidative stress, inflammation and apoptosis in microglia. Accordingly, LRRK2 deletion or inhibition of LRRK2-MAPK signaling attenuates Mn toxicity, suggesting that Mn toxicity is mediated at least in part by the activation of LRRK2-MAPK signaling. These events describe the role of LRRK2 in Mn-induced microglial toxicity which may lead to neuroinflammation. ↑ (blue), effects of Mn; ↓ & ⊥ (red), effects of LRRK2 KO and the LRRK2 kinase inhibitor GSK.

Taken together, our findings suggest that LRRK2 kinase activity contributes to Mn-induced oxidative stress, inflammation and apoptosis via activation of MAPK p38 and ERK in microglia (Fig 11). Mn-induced ROS production via LRRK2 activation may elevate the levels of pro-inflammatory cytokines and activate pro-apoptotic signaling pathways, leading to cell death. Many potential LRRK2 substrates have been identified, including LRRK2 itself via autophosphorylation largely within the Roc domain [89, 90], β-tubulin, FoxO1, tau, endophilin A1 and multiple Rab GTPases [84, 89, 91–94]. This indicates that Mn may modulate some of the downstream proteins such as MAPKs by increasing LRRK2 kinase activity. Further investigation is necessary to understand how Mn modulates LRRK2, particularly its kinase activity, which appears to be involved in Mn-induced toxicity in microglia. Nonetheless, our findings that LRRK2 kinase plays an important role in Mn toxicity opens new avenues to explore therapeutic interventions to treat Mn toxicity, as well as other neurodegenerative diseases associated with aberrant LRRK2 function.

## Supporting information

S1 Fig Inhibition of LRRK2 attenuates MPP^+^-induced oxidative stress. (**A**) After cells (LRRK2 WT and KO RAW 264.7) were exposed to MPP^+^ (250 μM) for 10 h, ROS were measured by fluorometer using DCF-fluorescence reagent to determine oxidative stress as described in the Methods section. (**B**) After pre-treatment with GSK (1 μM) or MLi-2 (50 nM) for 90 min, LRRK2 WT RAW 264.7 cells were exposed to MPP^+^ (250 μM) for 10 h, followed by ROS measurement by a fluorometer. ***, *p* < 0.001; ^@@@^, *p* < 0.001; compared to the control (one-way ANOVA followed by Tukey’s post hoc test; n = 6). The data shown are representative of 3 independent experiments.
(TIF)
