# Expression of Concern: LRRK2 kinase plays a critical role in manganese-induced inflammation and apoptosis in microglia

**DOI:** 10.1371/journal.pone.0296050

**Published:** 2023-12-13

**Authors:** The PLOS ONE Editors

After this article [1] was published, concerns were raised about Figs 4 and 9.

Specifically:

- The LRRK2 KO β-actin panel in Fig 4A appears similar to the LRRK2 WT p38 panel in Fig 9A.

The authors stated that the above concern was due to errors made during preparation of the figures and stated that the panel in Fig 9A was correct. The authors provided the underlying panel and accompanying quantification data for Fig 4A (S1 File and S2 File) along with accompanying underlying data for Fig 9A (S3 File and S4 File). The above concern is therefore resolved.

In reviewing this matter, PLOS noted that the primary data were not provided with the published article [1] contrary to the Data Availability Statement. The authors stated that not all the underlying data for this article remain available, and therefore this article does not comply with the PLOS data availability policy.

In light of the above concern pertaining to data availability, the *PLOS ONE* Editors issue this Expression of Concern.

## Supporting information

S1 File Fig 4A β-actin uncropped blot. (PPTX)

S2 File Fig 4B Quantitative Data. (PZFX)

S3 File Fig 9A p-38 uncropped blot. (PPTX)

S4 File Fig 9A Quantitative Data. (PZFX)
