# Correction: LRRK2 kinase plays a critical role in manganese-induced inflammation and apoptosis in microglia

**DOI:** 10.1371/journal.pone.0300095

**Published:** 2024-03-01

**Authors:** Judong Kim, Edward Pajarillo, Asha Rizor, Deok-Soo Son, Jayden Lee, Michael Aschner, Eunsook Lee

Following the Expression of Concern [2] published on this article [1], the underlying data supporting the results of this article are provided by the authors in this correction.

The correct Data Availability statement is: The underlying quantitative data for all figures are located at https://figshare.com/articles/figure/Figures_for_PLOS_One_LRRK2_manuscript/25219439.

By providing these data, the data availability concerns presented in the Expression of Concern are fully resolved.
