# Correction: LRRK2 kinase plays a critical role in manganese-induced inflammation and apoptosis in microglia

**DOI:** 10.1371/journal.pone.0324491

**Published:** 2025-05-13

**Authors:** Judong Kim, Edward Pajarillo, Asha Rizor, Deok-Soo Son, Jayden Lee, Michael Aschner, Eunsook Lee

Following the Expression of Concern [2] and subsequent Correction [3] published on this article [1], concerns were raised regarding the interpretation of the flow cytometry results presented in Figs 2 and 3.

**Fig 2:**
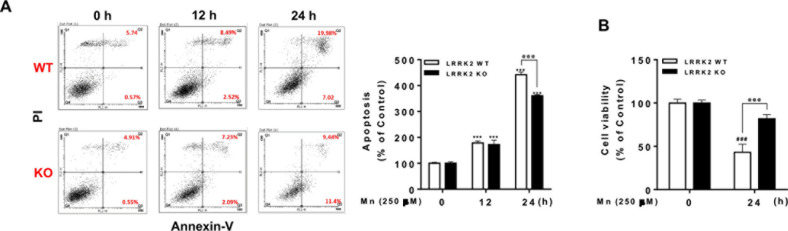
Deletion of LRRK2 attenuates Mn-induced apoptosis and cell death in RAW 264.7 cells. **(A)** LRRK2 WT or KO RAW 264.7 cells were treated with Mn (250 μM) for the designated times, followed by flow cytometry analysis to determine Mn-induced apoptosis. Both early (Q3) and late apoptotic/necrotic cells (Q2) were measured. **(B)** At the end of Mn exposure, cell viability was determined by MTT assay. ^@@@^, *p* < 0.001; ^###^, *p* < 0.001; ***, *p* < 0.001 compared to the control (one-way ANOVA followed by Tukey’s post hoc test; n = 3, for apoptosis assay; n = 6, for MTT assay). The data shown are representative of 3 independent experiments.

**Fig 3:**
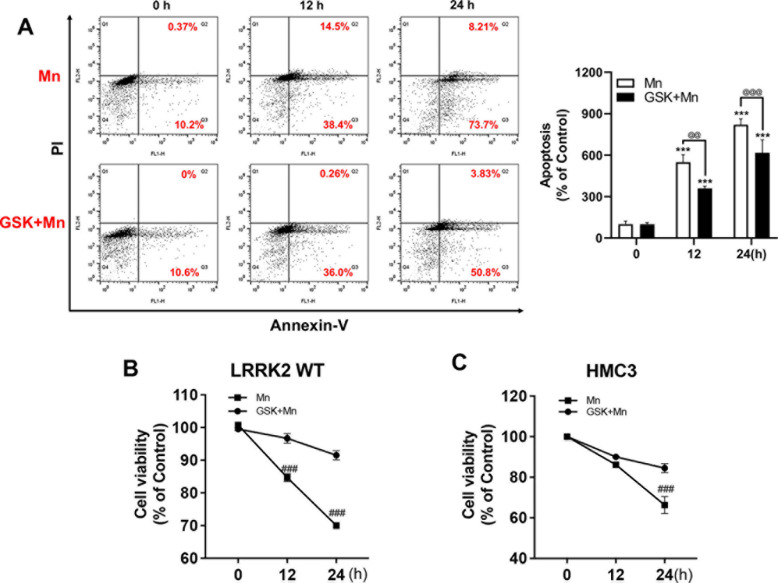
Inhibition of LRRK2 kinase activity attenuates Mn-induced apoptosis. **(A)** After pre-treatment with GSK (1 μM) for 90 min, cells (HMC3) were exposed to Mn (250 μM) for the designated time periods, followed by annexin V and PI staining and flow cytometry analysis to determine apoptosis. Early (Q3) and late apoptotic/necrotic cells (Q2) were analyzed. **(B, C)** After pre-treatment with GSK (1 μM) for 90 min, cells (LRRK2 WT RAW 264.7 and HMC3) were exposed to Mn for designated time periods, followed by the MTT assay to determine cell viability, as described in the Methods section, ^@@@^, *p* < 0.001; ^@@^, *p* < 0.01; ***, *p* < 0.001 compared to the control (one-way ANOVA followed by Tukey’s post hoc test; n = 3, apoptosis assay; n = 6, MTT assay). The data shown are representative of 3 independent experiments.

Specifically, it appears necrotic cells have been included in a count of apoptotic cells in the Q2 population (Annexin V+ PI+).

The authors comment that the flow cytometry machine and program used for the experiment presented the imaging data in a clockwise quadrant image arrangement; Q1 is top left, Q2 is top right, Q3 is bottom right, and Q4 is bottom left, which was not clearly annotated in the original article. Because of the missing quadrant annotation, the published figure legends for Figs 2 and 3 could be misinterpreted. With this notice, Figs 2 and 3 have been updated to include quadrant annotations, and the legends for these figures have been updated to clarify the correct quadrant references.

Additionally, in Fig 3, it was noted that the majority of the cell population falls in Q1 (dead cells/necrotic) cells, even without treatments (0 hrs of Mn2+ treatment). This is due to incorrect gating. This error is corrected in an updated Fig 3 provided here.

The authors’ explanation, the gating strategy (S1 File), and the underlying data provided for Figs 2 and 3 (S2–S4 Files) were reviewed by an independent member of the *PLOS One* Editorial Board and an independent FACS expert, who concluded that the data provided (S1–S4 Files) appear to support the published results.

The authors apologize for the errors in the published article.

## Supporting information

S1 File Gating strategy. (DOCX)

S2 File Figs 2 raw data. (ZIP)

S3 File Figs 3 raw data. (ZIP)

S4 File Figs 2 and 3 controls. (ZIP)
