# Supplementary figures and images for: LRRK2 kinase plays a critical role in manganese-induced inflammation and apoptosis in microglia
Source: PLoS One. 2019 Jan 15;14(1):e0210248. doi: 10.1371/journal.pone.0210248 (PMC6333340; doi:10.1371/journal.pone.0210248)

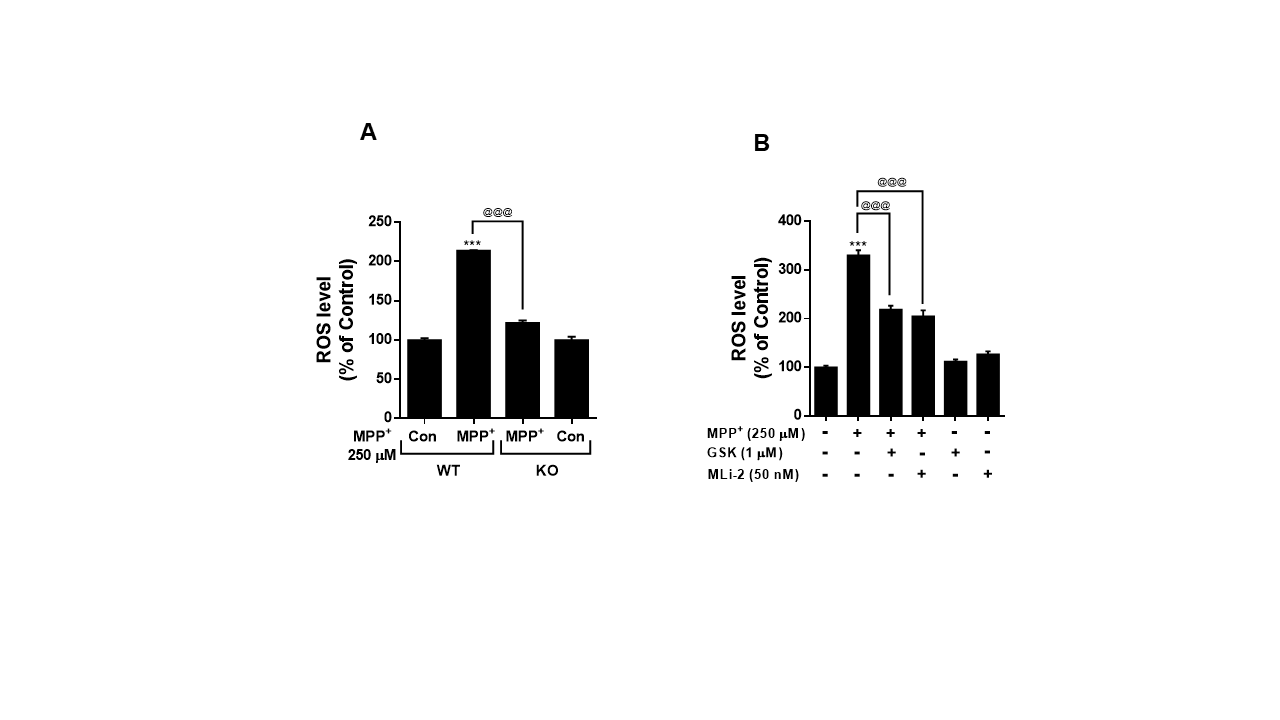

Supplement: S1 Fig — (A) After cells (LRRK2 WT and KO RAW 264.7) were exposed to MPP+ (250 μM) for 10 h, ROS were measured by fluorometer using DCF-fluorescence reagent to determine oxidative stress as described in the Methods section. (B) After pre-treatment with GSK (1 μM) or MLi-2 (50 nM) for 90 min, LRRK2 WT RAW 264.7 cells were exposed to MPP+ (250 μM) for 10 h, followed by ROS measurement by a fluorometer. ***, p < 0.001; @@@, p < 0.001; compared to the control (one-way ANOVA followed by Tukey’s post hoc test; n = 6). The data shown are representative of 3 independent experiments. (TIF) [file pone.0210248.s001.TIF]
