# Supplementary figures and images for: Expression of Concern: LRRK2 kinase plays a critical role in manganese-induced inflammation and apoptosis in microglia
Source: PLoS One. 2023 Dec 13;18(12):e0296050. doi: 10.1371/journal.pone.0296050 (PMC10718406; doi:10.1371/journal.pone.0296050)

## Slide 1
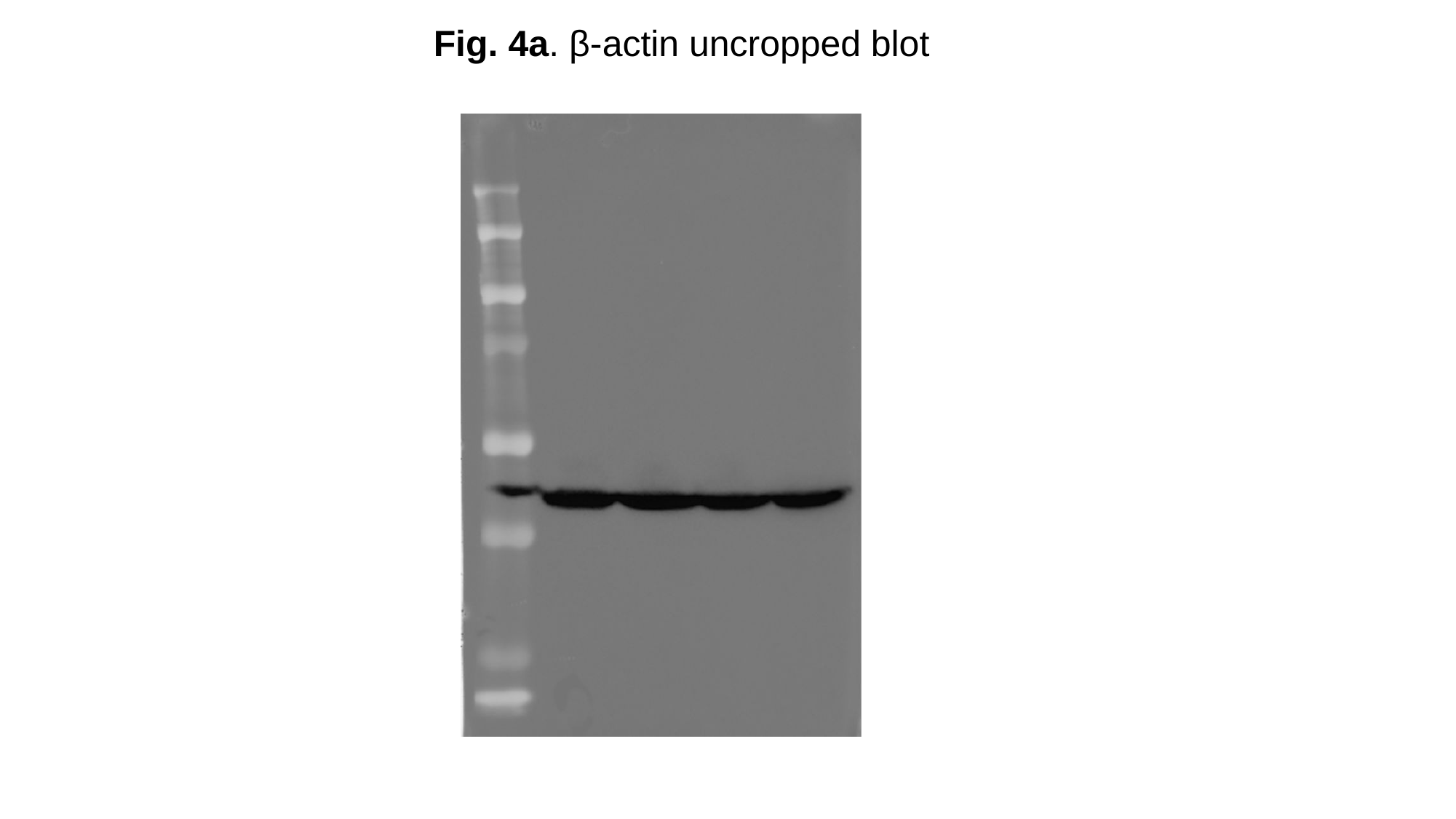

Fig. 4a. β-actin uncropped blot

Supplement: S1 File — (PPTX) [file pone.0296050.s001.pptx]

## Slide 1
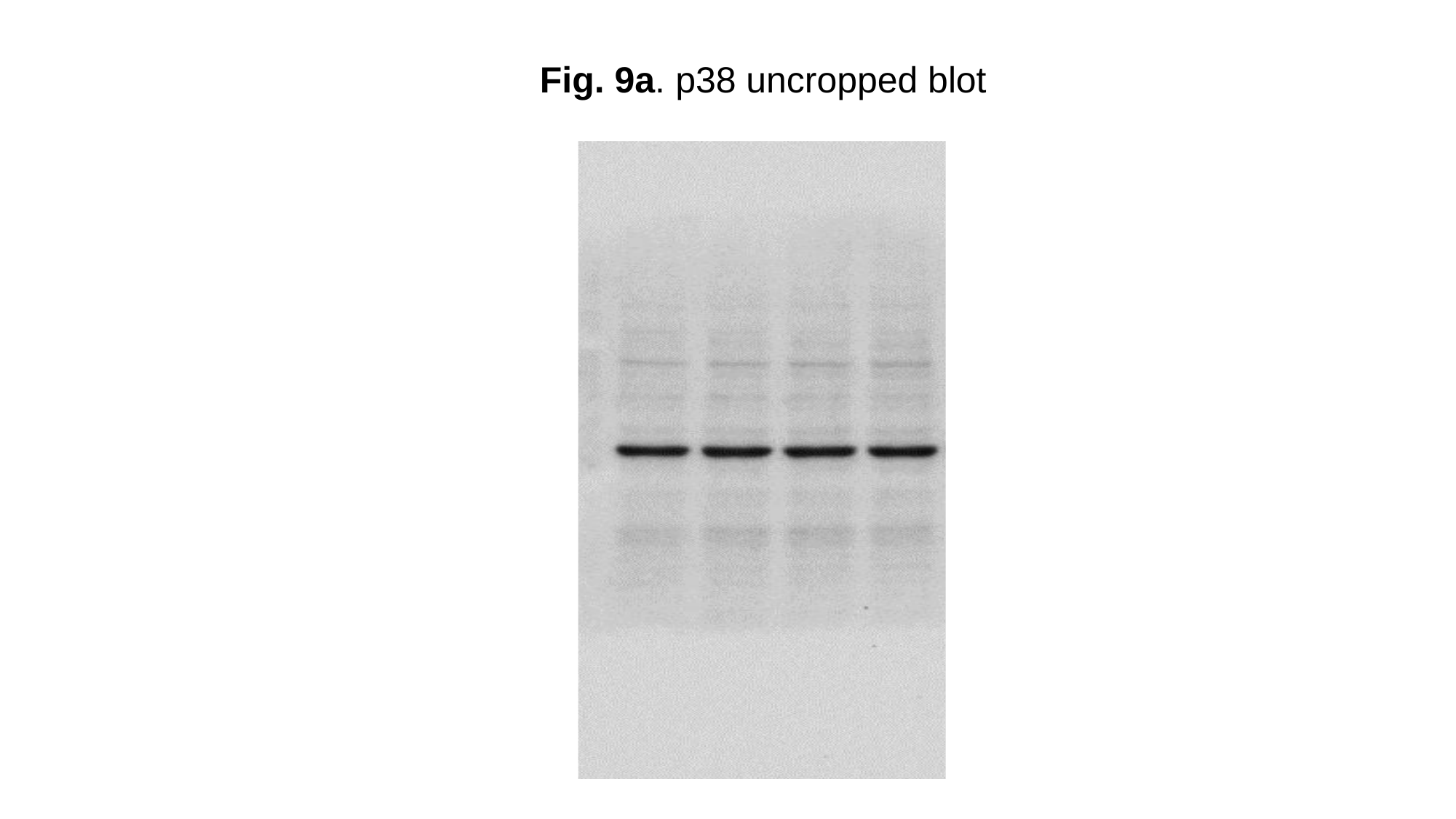

Fig. 9a. p38 uncropped blot

Supplement: S3 File — (PPTX) [file pone.0296050.s003.pptx]
