# Supplementary material for: Correction: LRRK2 kinase plays a critical role in manganese-induced inflammation and apoptosis in microglia
Source: PLoS One. 2025 May 13;20(5):e0324491. doi: 10.1371/journal.pone.0324491 (PMC12074335; doi:10.1371/journal.pone.0324491)
Supplement: S1 File — (DOCX) [file pone.0324491.s001.docx]

**Gating Strategy for Flow Cytometry Analysis of Apoptotic Cells Using Annexin V and Propidium Iodide (PI)**

**1. Excluding Debris from Samples**

- Create a forward scatter (FSC) vs. side scatter (SSC) plot to differentiate cells from debris.
- Exclude debris, typically found in the lower-left corner of the FSC vs. SSC plot.
- If only FSC-H and SSC-H are available (and not FSC-A or SSC-A), proceed with these parameters to differentiate cells.

**2. Set Up Compensation Controls**

- Use single-stained control samples labelled and stained with Annexin V+ only, PI+ only, and a sample of untreated and unstained cells - double-negative (Annexin V-/PI-) - to adjust for spectral overlap between fluorochromes.
- Ensure compensation is properly configured before analyzing experimental samples. Refine the debris exclusion gate based on this population.

**3. Setting up the Correct Axes for Apoptosis Analysis**

- Assign Annexin V to the X-axis and PI to the Y-axis in the analysis software for apoptosis evaluation.

**4. Identify Groups of Cell Populations Using Quadrants**

- Divide the Annexin V vs. PI plot into four quadrants to identify different cell states:
  - Q1 (Annexin V-/PI+): Necrotic cells
  - Q2 (Annexin V+/PI+): Late apoptotic cells
  - Q3 (Annexin V+/PI-): Early apoptotic cells
  - Q4 (Annexin V-/PI-): Live cells

**5. Focus on Relevant Quadrants**

- For treated samples, focus on Q2 (late apoptotic cells) and Q3 (early apoptotic cells) to evaluate apoptosis progression.
- Compare the distribution of cells in these quadrants between untreated controls and treated samples to assess the treatment effect.

**Additional Notes**

- This strategy applies to experiments using Annexin V and PI only, without additional live/dead staining kits.
- If single-cell discrimination is not possible due to missing FSC-A or SSC-A parameters, proceed directly with the Annexin V vs. PI plot, ensuring proper debris exclusion.
